# Associations of long-term exposure to air pollution, physical activity with blood pressure and prevalence of hypertension: the China Health and Retirement Longitudinal Study

**DOI:** 10.3389/fpubh.2023.1137118

**Published:** 2023-05-03

**Authors:** Jinglong Zhang, Fen Zhang, Chao Xin, Zhizhou Duan, Jing Wei, Xi Zhang, Shichao Han, Zhiping Niu

**Affiliations:** ^1^Department of Cardiovascular Surgery, Xijing Hospital, The Fourth Military Medical University, Xi'an, China; ^2^Department of Hepatobiliary Surgery, Xijing Hospital, The Fourth Military Medical University, Xi'an, China; ^3^PLA Rocket Force Characteristic Medical Center, Beijing, China; ^4^Preventive Health Service, Jiangxi Provincial People's Hospital, The First Affiliated Hospital of Nanchang Medical College, Nanchang, China; ^5^Department of Atmospheric and Oceanic Science, Earth System Science Interdisciplinary Center, University of Maryland, College Park, MD, United States; ^6^The First Clinical Medical College, Anhui Medical University, Hefei, Anhui, China; ^7^Department of Urology, Xijing Hospital, The Fourth Military Medical University, Xi'an, China

**Keywords:** air pollution, blood pressure, hypertension, physical activity, middle-aged and older adults, cardiovascular system

## Abstract

**Background:**

Long-term exposure to air pollution and physical activity (PA) are linked to blood pressure and hypertension. However, the joint effect of air pollution and PA on blood pressure and hypertension are still unknown in Chinese middle-aged and older adults.

**Methods:**

A total of 14,622 middle-aged and older adults from the China Health and Retirement Longitudinal Study wave 3 were included in this study. Ambient air pollution [particulate matter with diameter ≤ 2.5 μm (PM_2.5_), or ≤10 μm (PM_10_), sulfur dioxide (SO_2_), nitrogen dioxide (NO_2_), carbonic oxide (CO)] were estimated using satellite-based spatiotemporal models. PA was investigated using International Physical Activity Questionnaire. Generalized linear models were used to examine the associations of air pollution, PA score with blood pressure [systolic blood pressure (SBP), diastolic blood pressure (DBP), and mean arterial pressure (MAP)], and the prevalence of hypertension. Subgroup analysis was conducted to investigate the effects of air pollution on blood pressure and the prevalence of hypertension in different PA groups.

**Results:**

The results showed that for each inter-quartile range (IQR) increase in PM_2.5_ (25.45 μg/m^3^), PM_10_ (40.56 μg/m^3^), SO_2_ (18.61 μg/m^3^), NO_2_ (11.16 μg/m^3^), CO (0.42 mg/m^3^) and PA score (161.3 MET/h-week), the adjusted odd ratio (OR) of hypertension was 1.207 (95% confidence interval (CI): 1.137, 1.281), 1.189 (95%CI: 1.122, 1.260), 1.186 (95%CI: 1.112, 1.266), 1.186 (95%CI: 1.116, 1.260), 1.288 (95%CI: 1.223, 1.357), 0.948 (95%CI: 0.899, 0.999), respectively. Long-term exposure to PM_2.5_, PM_10_, SO_2_, NO_2_, and CO was associated with increased SBP, DBP, and MAP levels. For example, each IQR increase in PM_2.5_ was associated with 1.20 mmHg (95%CI: 0.69, 1.72) change in SBP, 0.66 mmHg (95%CI: 0.36, 0.97) change in DBP, and 0.84 mmHg (95%CI: 0.49, 1.19) change in MAP levels, respectively. Each IQR increase in PA score was associated with −0.56 mmHg (95%CI: −1.03, −0.09) change in SBP, −0.32 mmHg (95%CI: −0.59, −0.05) change in DBP, and −0.33 mmHg (95%CI: −0.64, −0.02) change in MAP levels, respectively. Subgroup analysis found that the estimated effects in the sufficient PA group were lower than that in the insufficient PA group.

**Conclusion:**

Long-term exposure to air pollutants is associated with increased blood pressure and hypertension risk, while high-level PA is associated with decreased blood pressure and hypertension risk. Strengthening PA might attenuate the adverse effects of air pollution on blood pressure and hypertension risk.

## 1. Introduction

Hypertension has become the top leading risk factor for attributable death, accounting for ~10.8 million deaths each year (1). Moreover, hypertension is one of the most significant risk factors for other cardiovascular diseases (CVDs) and always leads to organs damage and dysfunction of the organs, such as the heart, brain, and kidney (2–4). Previous studies showed that both blood pressure and the risk of hypertension increase significantly with age (5), and middle-aged and older adults were identified as the population at high risk for hypertension and other CVDs (3, 6). With the deepening aging of society, the burden of hypertension and hypertension-related diseases will greatly increase in the next few decades (1). Therefore, it is critical to identify the risk factor of hypertension and further explore its intervention strategies for the health promotion of middle-aged and older adults.

Several studies have indicated that exposure to ambient air pollution was associated with increased blood pressure and the prevalence of hypertension (8–11). A systematic review and meta-analysis indicated that long-term exposure to PM_2.5_, PM_10_, and NO_2_ was associated with increased diastolic blood pressure (DBP) levels, and PM_2.5_ was associated with an increased prevalence of hypertension (11). A cross-sectional study in rural China found that exposure to PM_2.5_, PM_10_, and NO_2_ was associated with increased SBP, DBP (except for PM_10_), mean arterial pressure (MAP) and the prevalence of hypertension (7). However, some studies have reported opposite results or insignificant associations (12). For example, a longitudinal study from the Jackson Heart Study found negative associations between PM_2.5_ and blood pressure, while no significant association between PM_2.5_ and hypertension was observed (12). Therefore, no consistent conclusion was reached, and evidence from middle-aged and older adults with higher CVD risks was still scarce. Moreover, most of the published studies were focused on the effects of particulate matter (PM_2.5_, PM_10_) and NO_2_, further studies are needed to determine whether exposure to other air pollutants (such as SO_2_, CO) can increase blood pressure and hypertension risk.

Physical activity (PA) has been demonstrated as an effective way against hypertension and other CVDs (13–15).

Numerous studies have found that higher PA levels could reduce blood pressure levels and hypertension risk (16–18). A meta-analysis of cohort studies found that each 10 metabolic equivalents of task (MET) hour/week increase in PA was associated with a 6% decrease in hypertension risk (16). After Liu et al.’s study, a longitudinal study of 12,511 adults from the China Health and Nutrition Survey found that people with higher PA levels had lower hypertension risk and blood pressure (SBP and DBP) levels (18). However, some studies reported inconsistent results (19, 20). For example, a national cross-section study of 18,231 Malaysian adults found that PA level was positively associated with SBP levels, whereas no significant association was observed for DBP level (20). In addition to the uncertainty of results, the majority of studies focused on the separate effect of PA, and litter attention was paid to the effects of PA in different environmental conditions, such as air pollution.

It cannot be ignored that PA also increases the risk of exposure to higher air pollution and leads to higher inhaled air pollutants, which might reduce or even negate the benefits of PA (21). Several studies have examined the joint effects of air pollution and PA on blood pressure (21, 22). For example, a nationwide cohort study of the Prediction for Atherosclerotic Cardiovascular Disease Risk in China (China-PAR) examined the modification effect of long-term exposure to PM_2.5_ in the associations between PA and hypertension incidence and indicated that PA was associated with decreased hypertension only among participants with low PM_2.5_ exposure (22). As for blood pressure, a cohort study of the China Health and Retirement Longitudinal Study (CHARLS) found that exposure to higher PM_2.5_ could attenuate the beneficial effects of PA on blood pressure (21). However, those two studies only examined the modification effects of PA in the associations of air pollution with hypertension and/or blood pressure, the health effects of air pollution on blood pressure and hypertension risk among different PA groups have still not been investigated. Moreover, to our knowledge, those published studies focused on PM_2.5_, the effects of other air pollutants among different PA groups have not been examined, especially gaseous pollutants. Further studies with more air pollutants are warranted to provide more comprehensive evidence of the adverse effects of air pollution on blood pressure and hypertension among different PA groups.

In this study, we aimed to examine the associations of air pollution (PM_2.5_, PM_10_, SO_2_, NO_2_, and CO) and PA with three blood pressure components (SBP, DBP, and MAP) and the prevalence of hypertension, and investigate the effects of air pollution on blood pressure and prevalence of hypertension in different PA groups.

## 2. Methods

### 2.1. Study population

This study used the data from the China Health and Retirement Longitudinal Study (CHARLS), which is established to collect a wide range of high microdata of middle-aged and older adults. Detailed description of the CHARLS study has been reported in previous studies (23–25). Briefly, a nationally representative sample of Chinese adults who aged 45 years or older was recruited using a four-stage stratified probability sampling method and participants were recruited from 450 communities, 150 county-level cities in 28 provinces. Four nationwide surveys were conducted in 2011, 2013, 2015, and 2018, respectively. Since blood pressure was only examined in 2011 and 2015, and the estimation of air pollution was mainly performed after 2013, we conducted a cross-sectional study of the CHARLS study wave 3 in 2015. A total of 16,406 participants completed blood pressure examinations. After excluding participants who aged below 45 years, without 3-time blood pressure measurements or air pollution data, 14,622 participants were included in the study.

All participants signed informed consent and the CHARLS was approved by the Institutional Review Board of Peking University (Code: IRB00001052-11015).

### 2.2. Assessment of air pollution

The concentrations of PM_2.5_, PM_10_, SO_2_, NO_2_, and CO were estimated at 0.1 degree (≈10 km) with space–time extremely randomized trees models using ground-based measurements, remote sensing products, and atmospheric re-analysis, and are collected from the ChinaHighAirPollutants (CHAP) dataset.^1^ The cross-validation *R*^2^ (root-mean-square error, RMSE) were 0.92 (10.76 μg/m^3^), 0.90 (21.12 μg/m^3^), 0.84 (10.07 μg/m^3^), 0.84 (7.99 μg/m^3^) and 0.80 (0.29 mg/m^3^) for daily PM_2.5_, PM_10_, SO_2_, NO_2_, and CO, respectively. Detailed information of the air pollution estimation is described in our previous study (26–29). Annual concentrations of air pollutants of each individual were matched according to their residential cities. Three-year average concentrations of air pollutants were defined as long-term exposure for the main effect models (7, 30, 31), while 2-year average was used in the sensitivity analysis.

### 2.3. Assessment of PA

PA was estimated using International Physical Activity Questionnaire (IPAQ) via face-to-face interviews. Briefly, the frequency and duration of different intensities activity (including vigorous, moderate, and light intensity) during a week were investigated. PA duration score was calculated as the frequency (times/week) × duration (hour/time). And then PA score was assessed using metabolic equivalent (MET) a: PA score = 8.0 × vigorous PA duration +4.0 × moderate PA duration +3.3 × light PA duration (32, 33). According to the World Health Organization (WHO) recommendation on PA for adults, participants were further categorized into “sufficient PA group (total score of vigorous and moderate PA ≥600 MET-hour/week)” and “insufficient PA group (total score of vigorous and moderate PA <600 MET-hour/week)” (34, 35).

### 2.4. Blood pressure measurement and definition of hypertension

Three-time SBP and DBP measurements were completed using electronic blood pressure monitors (HEM-7200 Monitor, Omron, Japan). Participants were asked not to eat, exercise, smoke or drink alcohol within 30 min before the blood pressure measurement. MAP was defined as DBP + 1/3 (SBP-DBP) (36). The three-time average blood pressure was used in statistical analysis. Hypertension individual was defined as a participant with SBP ≥ 140 mmHg or DBP ≥ 90 mmHg, having clinical-diagnosed hypertension, or taking anti-hypertension medicine (7, 37).

### 2.5. Covariates

Numerous potential confounders were included in our study according to previous studies of long-term exposure to air pollution with blood pressure and/or hypertension, including meteorological factors (38–40), sociodemographic variables, socioeconomic variables and health behaviors (7, 41–43). Meteorological factors (temperature and relative humidity) data were available from the China Meteorological Administration.^2^ Sociodemographic (age, sex), socioeconomic variables (residence, education, marital status), and health behaviors (smoking and drinking status) were collected via face-to-face interviews (8, 7, 37, 40, 43). Education level was categorized into “elementary school or below,” “middle, high, vocational school and associate degree” and “college and above.” Marital status was classified into “married and living with a spouse” “married but living without a spouse” or “single, divorced, and widowed” groups. Smoking status was classified into “smoker” and “non-smoker.” Drinking status was categorized into “non-drinker,” “drink but less than once a month” and “drink more than once a month”(6).

### 2.6. Statistical analysis

The characteristics of participants were described as mean ± standard deviation (SD) for continuous variables and counts (percentage, %) were described for categorical variables, respectively. The difference of continuous variables between the hypertension group and non-hypertension group was tested using Student’s *t*-test, and the distribution discrepancy of categorical variables between the hypertension group and non-hypertension group was examined using Chi-square test (33, 44). The rate in parentheses was represented as percentage of different subgroups in total, non-hypertension, and hypertension groups, respectively.

Generalized linear models (GLMs) were performed to examine the associations of air pollution with blood pressure and the prevalence of hypertension. The results were presented as change in mmHg for blood pressure and odd ratio (OR) for hypertension per inter-quartile range (IQR) increase in air pollutants. We first performed crude model without any adjustments. Then, temperature and relative humidity were adjusted in adjusted model 1. Finally, sociodemographic, socioeconomic variables, and health behaviors were additionally adjusted in adjusted model 2.

To further explore the effect of PA on blood pressure/hypertension prevalence and the modification effects of PA in the associations of air pollution with blood pressure and the prevalence of hypertension, a two-stage analysis was developed. We first used GLM models to examine the associations of PA score with blood pressure and prevalence of hypertension. Then, subgroup analysis was performed to examine whether PA score could modify the adverse effects of air pollution on blood pressure and prevalence of hypertension by including an interaction term of air pollution and different PA groups (Sufficient PA group *v.s.* Insufficient PA group) (31, 32).

Sensitivity analysis was also performed to evaluate the robustness of our results based on the full-adjusted model. Firstly, we examined the association of 2-year exposure with blood pressure and the prevalence of hypertension. Then, we excluded participants who were taking anti-hypertension medicine and evaluated whether the associations of air pollution with blood pressure were responsible for anti-hypertension medicine (7, 12, 45). Finally, we performed generalized linear mixed models (GLMMs) and included community ID as a random effect term in the models to adjust the different lifestyle characteristics in different regions.

All the statistical analyses were performed by R version 4.1.2 and the missing data for PA was imputed by using “mice” package (32, 33). A two-tailed *p*-value < 0.05 was set as statistical significance.

## 3. Results

### 3.1. Descriptive statistics

**Figure 1:**
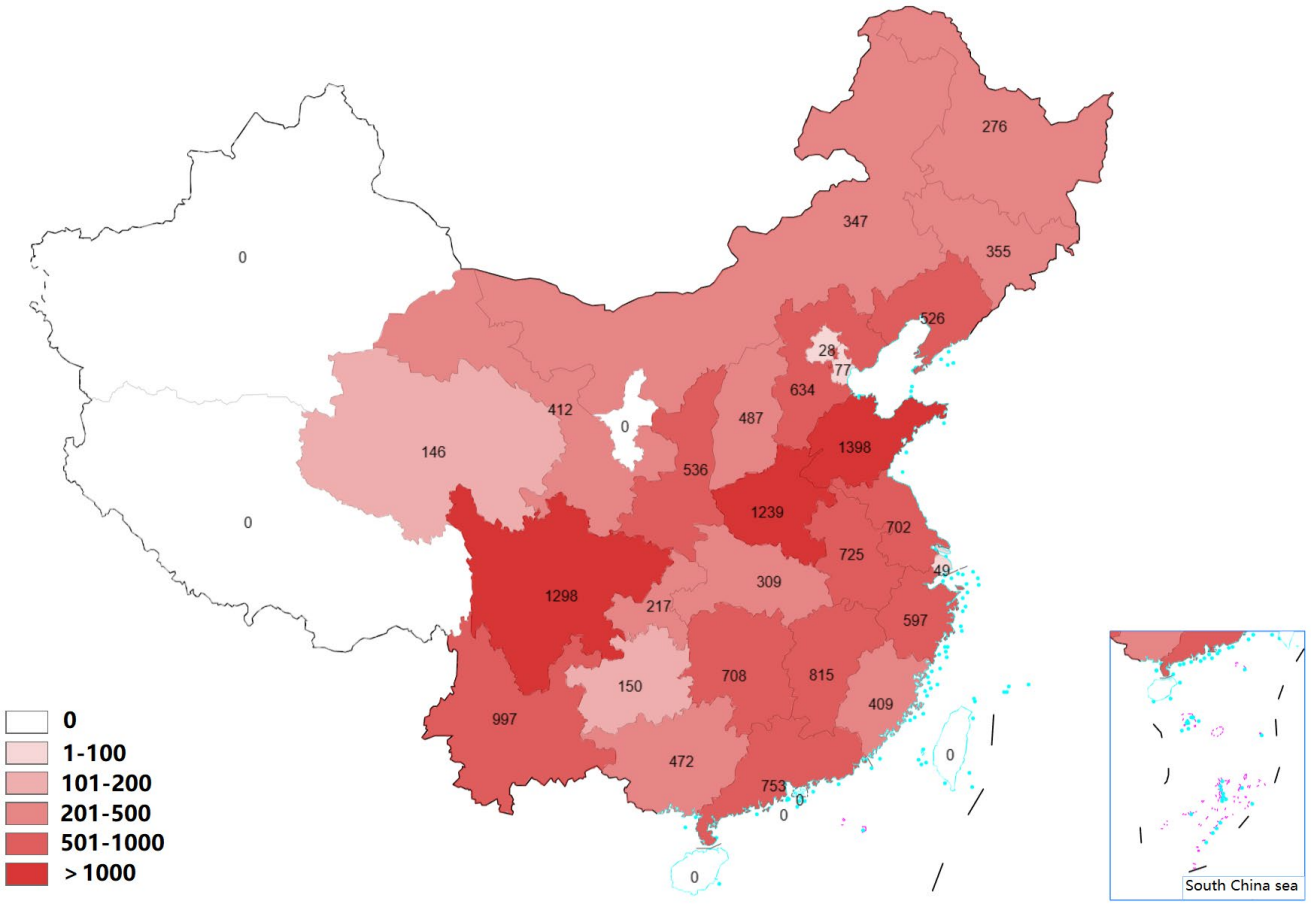
The geographical distribution of participants.

A total of 14,622 participants from 430 communities in 120 county-level cities were included in this study. The distribution of participants in 27 Chinese provinces is shown in Figure 1. A total of 5,191 participants were identified as hypertension cases, with a prevalence of 35.50%. A higher prevalence of hypertension was observed in the insufficient PA group than sufficient PA group. More detailed information about participants is shown in Table 1.

**Table 1:** Basic characteristics of the study population.

Characteristics^a^	Total (*n* = 14,662)	Non-hypertension (*n* = 9,471)	Hypertension (*n* = 5,191)	*p*-value^b^
**Age, years**	60.38 ± 10.05	58.59 ± 9.67	63.64 ± 9.93	<0.001
**Age group**				<0.001
≥65 years	9,833 (67.1)	2,479 (26.2)	2,350 (45.3)	
**Sex^c^**				0.121
Male	6,834 (46.6)	4,351 (45.9)	2,483 (47.8)	
**Residence**				<0.001
Rural	9,074 (61.9)	5,960 (62.9)	3,114 (60.0)	
**Education** ^**d**^				<0.001
Elementary school or below	2,564 (17.5)	1,620 (22.9)	944 (22.2)	
Middle, High, Vocational school and associate degree	5,427 (37.0)	3,154 (44.7)	2,273 (50.6)	
College and above	3,508 (23.9)	2,255 (31.9)	1,253 (27.9)	
**Marital status**				<0.001
Married and living with a spouse	12,047 (82.2)	7,964 (84.1)	4,083 (78.7)	
Married but living without a spouse	678 (4.6)	472 (5.0)	206 (4.0)	
Single, divorced, and widowed	1937 (13.2)	1,035 (10.9)	902 (17.4)	
**Smoking status** ^**e**^				<0.001
Non-smoker	10,023 (68.4)	5,825 (61.5)	4,197 (80.9)	
**Drinking status** ^**f**^				0.009
Non-drinker	3,899 (26.6)	2,451 (25.9)	1,448 (27.9)	
Drink but less than once a month	1,271 (8.7)	805 (8.5)	466 (9.0)	
Drink more than once a month	9,484 (64.7)	6,211 (65.6)	3,273 (63.1)	
**PA categories**				<0.001
Sufficient PA	7,754 (53.0)	5,293 (55.9)	2,461 (47.4)	

a Mean ± standard deviation for continuous variable, and count (percentage) for categorical variables. The rate in parentheses was represented as percentage of different subgroups in total, non-hypertension, and hypertension participants, respectively.

b *p*-value for the mean difference of continuous variable between non-hypertension participants and hypertension participants using Student’s *t*-test, and the distribution discrepancy of categorical variables between the hypertension group and non-hypertension group using Chi-square test.

c 146 missing data existed in sex.

d 3,132 missing data existed in education.

e 32 missing data existed in smoking status.

f 32 missing data existed in drinking status.

Table 2 presents the descriptive statistics of blood pressure, air pollution, and PA score. The average levels of SBP, DBP, MAP was 128.46 ± 19.86 mmHg, 75.42 ± 11.38 mmHg, 93.10 ± 13.14 mmHg, respectively. Three-year average concentrations of PM_2.5_, PM_10_, SO_2_, NO_2_, CO were 61.87 ± 18.60 μg/m^3^, 103.13 ± 32.34 μg/m^3^, 33.74 ± 14.07 μg/m^3^, 31.94 ± 7.61 μg/m^3^, 1.28 ± 0.38 mg/m^3^, respectively. Person correlation analysis found high co-linearity among different air pollutants (Supplementary Table S1). The average level of PA was 127.07 ± 108.95 MET/h-week.

**Table 2:** Descriptive statistics of blood pressure, air pollution, temperature, relative humidity, and PA score.

Variables	Mean	SD	P25	P50	P75	IQR
Blood pressure
SBP (mmHg)	128.46	19.86	114.00	126.00	140.00	26.00
DBP (mmHg)	75.42	11.38	67.33	74.67	82.67	15.34
MAP (mmHg)	93.10	13.14	83.67	92.11	101.33	17.66
Air pollutants
PM_2.5_ (μg/m^3^)	61.87	18.60	48.64	59.80	74.09	25.45
PM_10_ (μg/m^3^)	103.13	32.34	79.77	99.15	120.33	40.56
SO_2_ (μg/m^3^)	33.74	14.07	23.92	28.27	42.53	18.61
NO_2_ (μg/m^3^)	31.94	7.61	25.87	30.52	37.03	11.16
CO (mg/m^3^)	1.28	0.38	1.01	1.13	1.43	0.42
Meteorological factor	
Temperature (°C)	15.49	4.20	13.87	16.10	18.03	4.16
Relative Humidity (%)	67.49	9.83	61.33	70.00	75.00	13.67
Physical activity (PA)	
PA score (MET/h-week)	127.07	108.95	35.00	92.40	196.31	161.31

### 3.2. Associations of air pollution with blood pressure and prevalence of hypertension

Higher PM_2.5_, PM_10_, SO_2_, NO_2_, and CO exposure were associated with increased SBP, DBP and MAP in both crude model and adjusted models (Table 3). After adjusting for potential confounding factors, each IQR increase in PM_2.5_, PM_10_, SO_2_, NO_2_, and CO was associated with 1.20 mmHg (95%CI: 0.69, 1.72), 1.09 mmHg (95%CI: 0.58, 1.59), 1.81 mmHg (95%CI: 1.25, 2.38), 1.05 mmHg (95%CI: 0.52, 1.57), 1.44 mmHg (95%CI: 0.98, 1.89) increase in SBP, respectively. Each IQR increase in PM_2.5_, PM_10_, SO_2_, NO_2_, and CO was associated with 0.66 mmHg (95%CI: 0.36, 0.97), 0.56 mmHg (95%CI: 0.26, 0.85), 0.79 mmHg (95%CI: 0.45, 1.12), 0.54 mmHg (95%CI: 0.24, 0.85), 0.76 mmHg (95%CI: 0.50, 1.03) increase in DBP, respectively. And each IQR increase in PM_2.5_, PM_10_, SO_2_, NO_2_ and CO was associated with 0.84 mmHg (95%CI: 0.49, 1.19), 0.73 mmHg (95%CI: 0.39, 1.08), 1.13 mmHg (95%CI: 0.74, 1.52), 0.71 mmHg (95%CI: 0.35, 1.06), 0.99 mmHg (95%CI: 0.68, 1.29) increase in MAP, respectively.

**Table 3:** Associations of air pollution with blood pressure and prevalence of hypertension.

Air pollutants (IQR)	Blood pressure (mmHg and 95%CI)			Hypertension
	SBP	DBP	MAP	OR and 95%CI
PM_2.5_ (25.45 μg/m^3^)
Crude model	1.34 (0.90, 1.78)^***^	0.89 (0.64, 1.14)^***^	1.04 (0.75, 1.33)^***^	1.227 (1.172, 1.285)^***^
Adjusted model 1	1.28 (0.75, 1.81)^***^	0.60 (0.30, 0.91)^***^	0.83 (0.48, 1.18)^***^	1.190 (1.125, 1.259)^***^
Adjusted model 2	1.20 (0.69, 1.72)^***^	0.66 (0.36, 0.97)^***^	0.84 (0.49, 1.19)^***^	1.207 (1.137, 1.281)^***^
PM_10_ (40.56 μg/m^3^)
Crude model	1.15 (0.74,1.55)^***^	0.97 (0.74, 1.20)^***^	1.03 (0.76, 1.30)^***^	1.213 (1.163, 1.265)^***^
Adjusted model 1	1.13 (0.61, 1.66)^***^	0.53 (0.23, 0.83)^***^	0.73 (0.38, 1.08)^***^	1.175 (1.112, 1.242)^***^
Adjusted model 2	1.09 (0.58, 1.59)^***^	0.56 (0.26, 0.85)^***^	0.73 (0.39, 1.08)^***^	1.189 (1.122, 1.260)^***^
SO_2_ (18.61 μg/m^3^)
Crude model	1.54 (1.12, 1.97)^***^	1.21 (0.97, 1.46)^***^	1.32 (1.04, 1.60)^***^	1.226 (1.173, 1.282)^***^
Adjusted model 1	1.95 (1.36, 2.54)^***^	0.84 (0.50, 1.18)^***^	1.21 (0.82, 1.60)^***^	1.212 (1.140, 1.290)^***^
Adjusted model 2	1.81 (1.25, 2.38)^***^	0.79 (0.45, 1.12)^***^	1.13 (0.74, 1.52)^***^	1.186 (1.112, 1.266)^***^
NO_2_ (11.16 μg/m^3^)
Crude model	1.46 (0.99, 1.93)^***^	0.89 (0.62, 1.16)^***^	1.08 (0.77, 1.39) ^***^	1.254 (1.193, 1.317) ^***^
Adjusted model 1	1.35 (0.81, 1.89)^***^	0.58 (0.27, 0.89)^***^	0.84 (0.48, 1.19) ^***^	1.226 (1.158, 1.299) ^***^
Adjusted model 2	1.05 (0.52, 1.57)^***^	0.54 (0.24, 0.85)^***^	0.71 (0.35, 1.06) ^***^	1.186 (1.116, 1.260) ^***^
CO (0.42 mg/m^3^)
Crude model	1.04 (0.68, 1.40)^***^	0.89 (0.68, 1.09)^***^	0.94 (0.70, 1.17) ^***^	1.179 (1.135, 1.223)^***^
Adjusted model 1	1.17 (0.70, 1.64)^***^	0.65 (0.39, 0.92)^***^	0.82 (0.52, 1.13) ^***^	1.193 (1.136, 1.253) ^***^
Adjusted model 2	1.44 (0.98, 1.89)^***^	0.76 (0.50, 1.03)^***^	0.99 (0.68, 1.29) ^***^	1.288 (1.223, 1.357) ^***^

^***^ For *p*-value < 0.001.

Exposure to air pollution was associated with increased prevalence of hypertension. After fully adjusting for potential confounders, each IQR increase in PM_2.5_, PM_10_, SO_2_, NO_2_, and CO was associated with 20.7% (OR = 1.207, 95%CI: 1.137, 1.281), 18.9% (OR = 1.189, 95%CI: 1.122, 1.260), 18.6% (OR = 1.186, 95%CI: 1.112 1.266), 18.6% (OR = 1.186, 95%CI: 1.116, 1.260), 28.8% (OR = 1.288, 95%CI: 1.223, 1.357) increase in the prevalence of hypertension, respectively (Table 3).

### 3.3. Association of PA score with blood pressure and hypertension

PA score was negatively associated blood pressure and prevalence of hypertension. In the adjusted model, each IQR increase in PA score (161.3 MET/h-week) was associated with an −0.56 mmHg (95%CI: −1.03, −0.09), −0.32 mmHg (95%CI: −0.59, −0.05), −0.33 mmHg (95%CI: −0.64, −0.02) change in SBP, DBP and MAP, respectively. As for the prevalence of hypertension, each IQR increase in PA score was associated with a 5.2% (OR = 0.948, 95%CI:0.899, 0.999) decrease in the prevalence of hypertension (Table 4).

**Table 4:** Associations of PA score with blood pressure and the prevalence of hypertension.

Outcome	Crude model		Adjusted model	
	mmHg/OR and 95%CI	*p*-value	mmHg/OR and 95%CI	*p*-value
SBP (mmHg and 95%CI)	−0.80 (−1.27, −0.32)	0.001^**^	−0.56 (−1.03, −0.09)	0.019^*^
DBP (mmHg and 95%CI)	−0.21 (−0.48, 0.06)	0.133	−0.32 (−0.59, −0.05)	0.020^*^
MAP (mmHg and 95%CI)	−0.41 (−0.72, −0.09)	0.012^*^	−0.33 (−0.64, −0.02)	0.039^*^
Hypertension (OR and 95%CI)	0.893 (0.848, 0.939)	<0.001^***^	0.948 (0.899, 0.999)	0.048^*^

^*^For *p*-value < 0.05; ^**^for *p*-value < 0.01; ^***^for *p*-value < 0.001.

### 3.4. Subgroup analysis by different PA groups for the association of air pollution with blood pressure and hypertension

Exposure to air pollution was associated with increased blood pressure and prevalence of hypertension in both sufficient and insufficient PA group. When comparing the estimated effects in different PA groups, we found that the increased levels of blood pressure and prevalence were all lower in the sufficient PA group than that in insufficient PA group, even if the *P-interaction* was not significant (Table 5).

**Table 5:** Subgroup analysis by different PA groups for the associations of air pollution with blood pressure and prevalence of hypertension.

Air pollutants (IQR)	SBP		DBP		MAP		Hypertension	
	mmHg and 95%CI	P-_inter_	mmHg and 95%CI	P-_inter_	mmHg and 95%CI	P-_inter_	OR and 95%CI	P-_inter_
PM_2.5_ (25.45 μg/m^3^)		0.416		0.570		0.468		0.283
Sufficient PA group (*n* = 7,754)	1.01 (0.32, 1.70)^**^		0.58 (0.17, 0.98)^**^		0.72 (0.25, 1.19)^**^		1.176 (1.092, 1.268)^***^	
Insufficient PA group (*n* = 6,908)	1.36 (0.72, 2.00)^***^		0.72 (0.35, 1.10)^***^		0.93 (0.50, 1.37)^***^		1.241 (1.146, 1.343)^***^	
PM_10_ (40.56 μg/m^3^)		0.696		0.703		0.681		0.210
Sufficient PA group (*n* = 7,754)	1.00 (0.34, 1.66)^**^		0.50 (0.12, 0.89)^*^		0.67 (0.22, 1.12)^**^		1.157 (1.078, 1.243)^***^	
Insufficient PA group (*n* = 6,908)	1.15 (0.54, 1.77)^***^		0.59 (0.23, 0.95)^**^		0.78 (0.36, 1.20)^***^		1.225 (1.136, 1.321)^***^	
SO_2_ (18.61 μg/m^3^)		0.832		0.919		0.964		0.542
Sufficient PA group (*n* = 7,754)	1.85 (1.16, 2.54)^***^		0.77 (0.36, 1.17)^***^		1.12 (0.63, 1.60)^***^		1.169 (1.081, 1.265)^***^	
Insufficient PA group (*n* = 6,908)	1.76 (1.05, 2.48)^***^		0.79 (0.38, 1.21)^***^		1.13 (0.66, 1.60)^***^		1.204 (1.110, 1.305)^***^	
NO_2_ (11.16 μg/m^3^)		0.680		0.683		0.662		0.211
Sufficient PA group (*n* = 7,754)	0.93 (0.21, 1.65)^*^		0.47 (0.05, 0.90)^*^		0.62 (0.13, 1.12)^*^		1.149 (1.064, 1.241)^***^	
Insufficient PA group (*n* = 6,908)	1.12 (0.46, 1.78)^***^		0.58 (0.19, 0.97)^**^		0.76 (0.31, 1.21)^***^		1.229 (1.130, 1.335)^***^	
CO (0.42 mg/m^3^)		0.375		0.228		0.259		0.655
Sufficient PA group (*n* = 7,754)	1.27 (0.69, 1.84)^***^		0.63 (0.29, 0.97)^***^		0.84 (0.45, 1.23)^***^		1.276 (1.197, 1.361)^***^	
Insufficient PA group (*n* = 6,908)	1.58 (1.02, 2.14)^***^		0.88 (0.55, 1.20)^***^		1.11 (0.73, 1.49)^***^		1.299 (1.216, 1.389)^***^	

^*^For *p*-value < 0.05; ^**^for *p*-value < 0.01; ^***^for *p*-value < 0.001.

### 3.5. Sensitivity analysis

Except for associations of SO_2_ with DBP, MAP, and hypertension, the sensitivity analysis using 2-year average concentration of air pollutants showed consistent results (Supplementary Table S2). And the adverse effects of air pollution on blood pressure and the prevalence of hypertension were also observed after excluding the participants taking anti-hypertension medicine (Supplementary Table S3). Sensitivity analysis by including community ID as a random effect term in the GLMM models also showed similar results (except for PM_10_, and NO_2_ with DBP; Supplementary Table S4).

## 4. Discussion

In this study, we found that long-term exposure to ambient air pollutants was associated with increased blood pressure and the prevalence of hypertension. PA was associated with decreased blood pressure levels and prevalence of hypertension. While the *P-interactio*n values were not significant, subgroup analysis by different PA groups showed consistent trend that the estimated effects of air pollution on blood pressure and hypertension were all higher in the insufficient PA group than that in insufficient PA group, suggesting that PA might attenuate the adverse effects of air pollution on blood pressure and hypertension risk. Further studies are warranted to confirm our findings about the health effects of air pollution blood pressure and hypertension risk among people with different PA levels.

Our study suggested that exposure to air pollution was related to increased blood pressure levels and the prevalence of hypertension, which was in line with previous studies (7, 11, 46). For example, A cross-sectional study of 39,259 Chinese rural adults indicated that long-term exposure to PM_2.5_, PM_10_, and NO_2_ could significantly increase the prevalence of hypertension and blood pressure (SBP, DBP, and MAP), except for PM_10_ with SBP (7). Insignificant association between PM_10_ and SBP may be due to limited study regions, population characteristics, and chemical components of PM_10_. Additionally, we noticed that those studies were mainly focused on the effects of PM_2.5_, PM_10_, and NO_2_, litter attention was paid to other air pollutants. As important products of fossil fuel combustion, the health effects of exposure to SO_2_ and CO on blood pressure levels and hypertension should be noticeable (47). Our study indicated that long-term exposure to SO_2_ and CO may increase the prevalence of hypertension and blood pressure levels, including SBP, DBP, and MAP, which provided new evidence of adverse effects of SO_2_ and CO on cardiovascular system. Several potential biological mechanisms have been proposed to explain the adverse effects of air pollution on blood pressure and hypertension risk. Firstly, air pollution could increase oxidative stress, systemic inflammation and cause autonomic nervous system imbalance (11). Those pathways change would result from endothelial dysfunction (48), thrombotic pathway changes (49), epigenetic changes (50, 51), atherosclerosis (52), and activation of hypothalamic and pituitary–adrenal axis (HPA) (53) and further lead to hypertension risk and blood pressure levels increase (54).

PA is a well-established protective factor against chronic diseases. According to the WHO recommend, adults should take at least 75 min of vigorous PA or 150 min of moderate PA per week to reduce the risk of stroke, hypertension, diabetes and depression (55). Our study also found negative associations between PA with blood pressure and prevalence of hypertension, which was consistent with the WHO recommendation. Moreover, the benefits of PA on blood pressure and hypertension risk were also reported in previous studies (16, 17, 56). For example, a meta-analysis of 29 cohort studies found that each 10 MET/hour-week increase of PA was associated a 6% (relative risk = 0.94, 95%CI: 0.92, 0.96) decrease in hypertension risk (16). A meta-analysis of 15 randomized controlled trials (RCT) studies indicated that PA significantly reduced 24-h, day-time, and night-time blood pressure of hypertension patients (57). The benefits of PA on blood pressure and hypertension risk might be explained by reducing cardiac output, plasma norepinephrine levels, sympathetic activity, and total peripheral resistance (16, 21).

Our study investigated the effects of long-term exposure to air pollution on blood pressure and prevalence of hypertension in different PA groups and found that the adverse effects of blood pressure and prevalence were lower in the sufficient PA group than that in insufficient PA group. Despite no similar study has been published previously, our findings could be supported by the following aspects. Firstly, several studies investigated the modification effect of air pollution in the associations of PA with blood pressure and hypertension risk and indicated long-term exposure to air pollution could attenuate the beneficial effects of PA on blood pressure and hypertension risk (21, 22). Secondly, several studies reported that PA could attenuate the adverse effects of air pollution on chronic diseases, including metabolic syndrome (32, 58), diabetes (59), CVDs (60) and cognitive function (61). Thirdly, previous studies suggested that exposure to higher levels of air pollution could lead to oxidative stress, systemic inflammation and autonomic nervous system imbalance, and result in declines in immunity stability (62, 63). On the contrary, several studies indicated that PA could improve immune responses to lower chronic low-grade inflammation and improve immune markers in several disease conditions (62, 64, 65).

Some limitations should be mentioned in our study. Firstly, since blood pressure were only measured in 2011 and 2015, and air pollution was estimated after 2013 in our study, longitudinal study was not conducted. The cause-and-effect of the associations with blood pressure and hypertension still needs further research. Secondly, anti-hypertension medication use might also affect blood pressure. However, the results of excluding anti-hypertension treatment participants indicated that our findings were robust (7). Thirdly, muti-pollutant models were not developed due to the high co-linearity of air pollutants (66). Fourthly, similar to previous studies of long-term effects of air pollution on blood pressure, blood pressure was assessed using three-time average levels in single day, which might cause some bias. Further studies with precise measurement of the individual’s true blood pressure levels are needed to confirm our results. Fifthly, lifestyle and other potential confounders were not investigated in CHARLS, which might also influence blood pressure. However, in order to adjust the difference of lifestyle characteristics in different regions, we conducted sensitivity analysis by including the community ID as a random effect term and the results showed good consistency. Finally, although subgroup analysis by different PA groups showed consistent trends, the *P-interaction* was not significant. Further studies are warranted to confirm our results.

## 5. Conclusion

In conclusion, exposure to higher air pollution and lower PA are associated with increased blood pressure level and the prevalence of hypertension. The adverse effects of air pollution on blood pressure and prevalence of hypertension are lower in sufficient PA group than insufficient PA group, suggesting that PA might attenuate the adverse effects of air pollution on blood pressure and hypertension risk. Further studies are needed to confirm our findings about different effects of air pollution on blood pressure and hypertension among people with different PA levels.

## Data availability statement

The raw data supporting the conclusions of this article will be made available by the authors, without undue reservation.

## Ethics statement

All participants signed informed consents and the CHARLS was approved by the Institutional Review Board of Peking University (Code: IRB00001052-11015). The patients/participants provided their written informed consent to participate in this study.

## Author contributions

ZN, SH, and JZ: conceptualization. JZ, FZ, JW, and ZD: data curation. JZ, FZ, ZN, and ZD: formal analysis. SH: funding acquisition. JZ, FZ, CX, XZ, and ZD: investigation. ZN and SH: methodology, supervision, and writing–review and editing. JZ, FZ, CX, and XZ: project administration. JZ and ZN: resources. JZ and FZ: software and roles and writing–original draft. ZN: validation. JZ, FZ, and ZN: visualization. All authors contributed to the article and approved the submitted version.

## Funding

This study was supported by the National Natural Science Foundation of China (no. 82102322).

## Conflict of interest

The authors declare that the research was conducted in the absence of any commercial or financial relationships that could be construed as a potential conflict of interest.

## Publisher’s note

All claims expressed in this article are solely those of the authors and do not necessarily represent those of their affiliated organizations, or those of the publisher, the editors and the reviewers. Any product that may be evaluated in this article, or claim that may be made by its manufacturer, is not guaranteed or endorsed by the publisher.

## Footnotes

^1^Available at: https://weijing-rs.github.io/product.html.

^2^ http://www.cma.gov.cn/
