# Supplementary material for: Associations of long-term exposure to air pollution, physical activity with blood pressure and prevalence of hypertension: the China Health and Retirement Longitudinal Study
Source: Front Public Health. 2023 May 3;11:1137118. doi: 10.3389/fpubh.2023.1137118 (PMC10189054; doi:10.3389/fpubh.2023.1137118)
Supplement: Supplementary file 1 [file Data_Sheet_1.pdf]

Supplementary Material

Table S1. Person correlation coefficients of 3-year average air pollutant concentrations

**Air pollutants**	$PM_{2.5}$	PM_{10}	SO_2	NO_2	CO
$PM_{2.5}$	1				
PM_{10}	0.951***	1			
SO_2	0.785***	0.863***	1		
NO_2	0.882***	0.852***	0.744***	1	
CO	0.767***	0.814***	0.838***	0.738***	1

Abbreviations: $PM_{2.5}$, particle with aerodynamic diameter ≤2.5 μm; PM_{10}, particle with aerodynamic diameter ≤10 μm; SO_2, sulfur dioxide; NO_2, nitrogen dioxide; CO, carbonic oxide; * P<0.05; ** P<0.01; ***P<0.001.

Table S2. Sensitivity analysis by using 3-year average, 2-year average air pollution concentration in the associations of air pollution with blood pressure and hypertension.

Air pollutants	3-Year Average					2-Year Average				
	IQR	SBP (mmHg)	DBP (mmHg)	MAP (mmHg)	Hypertension (OR)	IQR	SBP (mmHg)	DBP (mmHg)	MAP (mmHg)	Hypertension (OR)
$PM_{2.5}$ (μg/m^3)	25.45	1.20 (0.69, 1.72) ***	0.66 (0.36, 0.97) ***	0.84 (0.49, 1.19) ***	1.207 (1.137, 1.281) ***	23.29	1.24 (0.73, 1.75) ***	0.67(0.37, 0.97) ***	0.86(0.51, 1.21) ***	1.205(1.007, 1.279) ***
PM_{10} (μg/m^3)	40.56	1.09 (0.58, 1.59) ***	0.56 (0.26, 0.85) ***	0.73 (0.39, 1.08) ***	1.189 (1.122, 1.260) ***	40.21	1.23(0.69, 1.78) ***	0.61(0.29, 0.93) ***	0.82(0.45, 1.19) ***	1.211(1.004, 1.290) ***
SO_2 (μg/m^3)	18.61	1.81(1.25, 2.38) ***	0.79 (0.45, 1.12) ***	1.13(0.74, 1.52) ***	1.186 (1.112, 1.266) ***	13.8	0.65(0.05, 1.24) *	0.08(-0.27, 0.43)	0.27(-0.14, 0.68)	1.066(1.000, 1.142)
NO_2 (μg/m^3)	11.16	1.05(0.52, 1.57) ***	0.54 (0.24, 0.85) ***	0.71 (0.35, 1.06) ***	1.186 (1.116, 1.260) ***	11.9	1.15(0.60, 1.69) ***	0.55(0.23, 0.87) ***	0.75(0.38, 1.11) ***	1.202(1.012, 1.280) ***
CO (mg/m^3)	0.42	1.44 (0.98, 1.89) ***	0.76 (0.50, 1.03) ***	0.99 (0.68, 1.29) ***	1.288 (1.223, 1.357) ***	0.37	1.31(0.88, 1.73) ***	0.69(0.44, 0.94) ***	0.89(0.60, 1.19) ***	1.275 (1.213, 1.340) ***

Notes: * P<0.05; ** P<0.01; ***P<0.001.

Table S3. Sensitivity analysis by including anti-hypertensive drug takers and excluding anti-hypertensive drug takers in the associations of air pollution with blood pressure and hypertension.

Air pollutants (IQR)	Drug tankers were included			Drug tankers were excluded		
	SBP	DBP	MAP	SBP	DBP	MAP
$PM_{2.5}$ (25.45 μg/m^3)	1.20 (0.69, 1.72) ***	0.66 (0.0.36, 0.97) ***	0.84 (0.49, 1.19) ***	1.00 (0.46, 1.54) ***	0.70 (0.37, 1.02) ***	0.80 (0.43, 1.17) ***
PM_{10} (40.56 μg/m^3)	1.09 (0.58, 1.59) ***	0.56 (0.26, 0.85) ***	0.73 (0.39, 1.08) ***	0.90 (0.38, 1.43) ***	0.58 (0.26, 0.90) ***	0.69 (0.32, 1.05) ***
SO_2 (18.61 μg/m^3)	1.81(1.25, 2.38) ***	0.79 (0.45, 1.12) ***	1.13(0.74, 1.52) ***	2.10 (1.51, 2.70) ***	1.05 (0.69, 1.41) ***	1.40 (0.99, 1.81) ***
NO_2 (11.16 μg/m^3)	1.05 (0.52, 1.57) ***	0.54 (0.24, 0.85) ***	0.71 (0.35, 1.06) ***	1.01 (0.46, 1.55) ***	0.67 (0.34, 1.00) ***	0.78 (0.41, 1.16) ***
CO (0.42 mg/m^3)	1.44 (0.98, 1.89) ***	0.76 (0.50, 1.03) ***	0.99 (0.68, 1.29) ***	1.31 (0.84, 1.78) ***	0.82 (0.53, 1.11) ***	0.98 (0.66, 1.31) ***

Notes: * P<0.05; ** P<0.01; ***P<0.001.

Table S4. Sensitivity analysis by including Community ID as random effect term

Air pollutants (IQR)	Community ID was not included as random effect term				Community ID was included as random effect term			
	SBP	DBP	MAP	Hypertension	SBP	DBP	MAP	Hypertension
$PM_{2.5}$ (25.45 μg/m^3)	1.20 (0.69, 1.72) ***	0.66 (0.36, 0.97) ***	0.84 (0.49, 1.19) ***	1.207 (1.137, 1.281) ***	1.13(0.29, 1.97) **	0.54(0.02, 1.06) *	0.74(0.14, 1.33) *	1.280(1.103, 1.324) ***
PM_{10} (40.56 μg/m^3)	1.09 (0.58, 1.59) ***	0.56 (0.26, 0.85) ***	0.73 (0.39, 1.08) ***	1.189 (1.122, 1.260) ***	0.96(0.13, 1.80) *	0.42(-0.10, 0.93)	0.60(0.01, 1.19) *	1.177(1.076, 1.288) ***
SO_2 (18.61 μg/m^3)	1.81(1.25, 2.38) ***	0.79 (0.45, 1.12) ***	1.13(0.74, 1.52) ***	1.186 (1.112, 1.266) ***	1.64(0.71, 2.57) ***	0.67(0.10, 1.25) *	0.99(0.34, 1.65) **	1.182(1.067, 1.308) **
NO_2 (11.16 μg/m^3)	1.05(0.52, 1.57) ***	0.54 (0.24, 0.85) ***	0.71 (0.35, 1.06) ***	1.186 (1.116, 1.260) ***	1.01(0.16, 1.86) *	0.46(-0.06, 0.98)	0.65(0.05, 1.25) *	1.185(1.080, 1.300) ***
CO (0.42 mg/m^3)	1.44 (0.98, 1.89) ***	0.76 (0.50, 1.03) ***	0.99 (0.68, 1.29) ***	1.288 (1.223, 1.357) ***	1.32(0.58, 2.06) ***	0.66(0.20, 1.11) ***	0.88(0.36, 1.40) **	1.291(1.194, 1.396) ***

Notes: * P<0.05; ** P<0.01; ***P<0.001.
