# Supplementary material for: Associations of long-term exposure to air pollution, physical activity with blood pressure and prevalence of hypertension: the China Health and Retirement Longitudinal Study
Source: Front Public Health. 2023 May 3;11:1137118. doi: 10.3389/fpubh.2023.1137118 (PMC10189054; doi:10.3389/fpubh.2023.1137118)
Supplement: Supplementary file 2 [file Table_1.DOCX]

**Supplementary Material**

**Table S1. Person correlation coefficients of 3-year average air pollutant concentrations**

| **Air pollutants** | PM_2.5_ | PM_10_ | SO_2_ | NO_2_ | CO |
| --- | --- | --- | --- | --- | --- |
| PM_2.5_ | 1 |  |  |  |  |
| PM_10_ | 0.951^***^ | 1 |  |  |  |
| SO_2_ | 0.785^***^ | 0.863^***^ | 1 |  |  |
| NO_2_ | 0.882^***^ | 0.852^***^ | 0.744^***^ | 1 |  |
| CO | 0.767^***^ | 0.814^***^ | 0.838^***^ | 0.738^***^ | 1 |

Abbreviations: PM_2.5_, particle with aerodynamic diameter ≤2.5 μm; PM_10_, particle with aerodynamic diameter ≤10 μm; SO_2_, sulfur dioxide; NO_2_, nitrogen dioxide; CO, carbonic oxide; * P<0.05; ** P<0.01; ***P<0.001.

**Table S2. Sensitivity analysis by using 3-year average, 2-year average air pollution concentration in the associations of air pollution with blood pressure and hypertension.**

| Air pollutants | 3-Year Average | | | | |  | 2-Year Average | | | | |
| --- | --- | --- | --- | --- | --- | --- | --- | --- | --- | --- | --- |
|  | IQR | SBP (mmHg) | DBP (mmHg) | MAP (mmHg) | Hypertension (OR) |  | IQR | SBP (mmHg) | DBP (mmHg) | MAP (mmHg) | Hypertension (OR) |
| PM_2.5_ (μg/m^3^) | 25.45 | 1.20 (0.69, 1.72) ^***^ | 0.66 (0.36, 0.97) ^***^ | 0.84 (0.49, 1.19) ^***^ | 1.207 (1.137, 1.281) ^***^ |  | 23.29 | 1.24 (0.73, 1.75) ^***^ | 0.67(0.37, 0.97) ^***^ | 0.86(0.51, 1.21) ^***^ | 1.205(1.007, 1.279) ^***^ |
| PM_10_ (μg/m^3^) | 40.56 | 1.09 (0.58, 1.59) ^***^ | 0.56 (0.26, 0.85) ^***^ | 0.73 (0.39, 1.08) ^***^ | 1.189 (1.122, 1.260) ^***^ |  | 40.21 | 1.23(0.69, 1.78) ^***^ | 0.61(0.29, 0.93) ^***^ | 0.82(0.45, 1.19) ^***^ | 1.211(1.004, 1.290) ^***^ |
| SO_2_ (μg/m^3^) | 18.61 | 1.81(1.25, 2.38) ^***^ | 0.79 (0.45, 1.12) ^***^ | 1.13(0.74, 1.52) ^***^ | 1.186 (1.112, 1.266) ^***^ |  | 13.8 | 0.65(0.05, 1.24) ^*^ | 0.08(-0.27, 0.43) | 0.27(-0.14, 0.68) | 1.066(1.000, 1.142) |
| NO_2_ (μg/m^3^) | 11.16 | 1.05(0.52, 1.57) ^***^ | 0.54 (0.24, 0.85) ^***^ | 0.71 (0.35, 1.06) ^***^ | 1.186 (1.116, 1.260) ^***^ |  | 11.9 | 1.15(0.60, 1.69) ^***^ | 0.55(0.23, 0.87) ^***^ | 0.75(0.38, 1.11) ^***^ | 1.202(1.012, 1.280) ^***^ |
| CO (mg/m^3^) | 0.42 | 1.44 (0.98, 1.89) ^***^ | 0.76 (0.50, 1.03) ^***^ | 0.99 (0.68, 1.29) ^***^ | 1.288 (1.223, 1.357) ^***^ |  | 0.37 | 1.31(0.88, 1.73) ^***^ | 0.69(0.44, 0.94) ^***^ | 0.89(0.60, 1.19) ^***^ | 1.275 (1.213, 1.340) ^***^ |

Notes: * P<0.05; ** P<0.01; ***P<0.001.

**Table S3. Sensitivity analysis by including anti-hypertensive drug takers and excluding anti-hypertensive drug takers in the associations of air pollution with blood pressure and hypertension.**

| Air pollutants (IQR) | Drug tankers were included | | |  | Drug tankers were excluded | | |
| --- | --- | --- | --- | --- | --- | --- | --- |
|  | SBP | DBP | MAP |  | SBP | DBP | MAP |
| PM_2.5_ (25.45 μg/m^3^) | 1.20 (0.69, 1.72) ^***^ | 0.66 (0.0.36, 0.97) ^***^ | 0.84 (0.49, 1.19) ^***^ |  | 1.00 (0.46, 1.54) ^***^ | 0.70 (0.37, 1.02) ^***^ | 0.80 (0.43, 1.17) ^***^ |
| PM_10_ (40.56 μg/m^3^) | 1.09 (0.58, 1.59) ^***^ | 0.56 (0.26, 0.85) ^***^ | 0.73 (0.39, 1.08) ^***^ |  | 0.90 (0.38, 1.43) ^***^ | 0.58 (0.26, 0.90) ^***^ | 0.69 (0.32, 1.05) ^***^ |
| SO_2_ (18.61 μg/m^3^) | 1.81(1.25, 2.38) ^***^ | 0.79 (0.45, 1.12) ^***^ | 1.13(0.74, 1.52) ^***^ |  | 2.10 (1.51, 2.70) ^***^ | 1.05 (0.69, 1.41) ^***^ | 1.40 (0.99, 1.81) ^***^ |
| NO_2_ (11.16 μg/m^3^) | 1.05 (0.52, 1.57) ^***^ | 0.54 (0.24, 0.85) ^***^ | 0.71 (0.35, 1.06) ^***^ |  | 1.01 (0.46, 1.55) ^***^ | 0.67 (0.34, 1.00) ^***^ | 0.78 (0.41, 1.16) ^***^ |
| CO (0.42 mg/m^3^) | 1.44 (0.98, 1.89) ^***^ | 0.76 (0.50, 1.03) ^***^ | 0.99 (0.68, 1.29) ^***^ |  | 1.31 (0.84, 1.78) ^***^ | 0.82 (0.53, 1.11) ^***^ | 0.98 (0.66, 1.31) ^***^ |

Notes: * P<0.05; ** P<0.01; ***P<0.001.

**Table S4. Sensitivity analysis by including Community ID as random effect term**

| Air pollutants (IQR) | Community ID was not included as random effect term | | | |  | Community ID was included as random effect term | | | |
| --- | --- | --- | --- | --- | --- | --- | --- | --- | --- |
|  | SBP | DBP | MAP | Hypertension |  | SBP | DBP | MAP | Hypertension |
| PM_2.5_ (25.45 μg/m^3^) | 1.20 (0.69, 1.72) ^***^ | 0.66 (0.36, 0.97) ^***^ | 0.84 (0.49, 1.19) ^***^ | 1.207 (1.137, 1.281) ^***^ |  | 1.13(0.29, 1.97) ^**^ | 0.54(0.02, 1.06) ^*^ | 0.74(0.14, 1.33) ^*^ | 1.280(1.103, 1.324) ^***^ |
| PM_10_ (40.56 μg/m^3^) | 1.09 (0.58, 1.59) ^***^ | 0.56 (0.26, 0.85) ^***^ | 0.73 (0.39, 1.08) ^***^ | 1.189 (1.122, 1.260) ^***^ |  | 0.96(0.13, 1.80) ^*^ | 0.42(-0.10, 0.93) | 0.60(0.01, 1.19) ^*^ | 1.177(1.076, 1.288) ^***^ |
| SO_2_ (18.61 μg/m^3^) | 1.81(1.25, 2.38) ^***^ | 0.79 (0.45, 1.12) ^***^ | 1.13(0.74, 1.52) ^***^ | 1.186 (1.112, 1.266) ^***^ |  | 1.64(0.71, 2.57) ^***^ | 0.67(0.10, 1.25) ^*^ | 0.99(0.34, 1.65) ^**^ | 1.182(1.067, 1.308) ^**^ |
| NO_2_ (11.16 μg/m^3^) | 1.05(0.52, 1.57) ^***^ | 0.54 (0.24, 0.85) ^***^ | 0.71 (0.35, 1.06) ^***^ | 1.186 (1.116, 1.260) ^***^ |  | 1.01(0.16, 1.86) ^*^ | 0.46(-0.06, 0.98) | 0.65(0.05, 1.25) ^*^ | 1.185(1.080, 1.300) ^***^ |
| CO (0.42 mg/m^3^) | 1.44 (0.98, 1.89) ^***^ | 0.76 (0.50, 1.03) ^***^ | 0.99 (0.68, 1.29) ^***^ | 1.288 (1.223, 1.357) ^***^ |  | 1.32(0.58, 2.06) ^***^ | 0.66(0.20, 1.11) ^***^ | 0.88(0.36, 1.40) ^**^ | 1.291(1.194, 1.396) ^***^ |

Notes: * P<0.05; ** P<0.01; ***P<0.001.
